# Long-term effects of the multidisciplinary risk assessment and management program for patients with diabetes mellitus (RAMP-DM): a population-based cohort study

**DOI:** 10.1186/s12933-015-0267-3

**Published:** 2015-08-14

**Authors:** Fangfang Jiao, Colman Siu Cheung Fung, Yuk Fai Wan, Sarah Morag McGhee, Carlos King Ho Wong, Daisy Dai, Ruby Kwok, Cindy Lo Kuen Lam

**Affiliations:** Department of Family Medicine and Primary Care, Li Ka Shing Faculty of Medicine, The University of Hong Kong, 3/F Ap Lei Chau Clinic, 161 Main Street, Ap Lei Chau, Hong Kong; School of Public Health, Li Ka Shing Faculty of Medicine, The University of Hong Kong, 5/F William MW Mong Block, 21 Sassoon Road, Pokfulam, Hong Kong; Primary and Community Services, Hospital Authority Head Office, Hong Kong Hospital Authority, Hospital Authority Building, 147B Argyle Street, Kowloon, Hong Kong

**Keywords:** Diabetes mellitus, Risk stratification, Multidisciplinary, Cardiovascular complications

## Abstract

**Background:**

Studies on the long-term effectiveness of multidisciplinary risk-stratification based management in Chinese population were rare. This study aimed to evaluate the effectiveness of a multidisciplinary risk assessment and management program for patients with diabetes mellitus (RAMP-DM) in reducing the risks of cardiovascular complications and all-cause mortality.

**Methods:**

A prospective cohort study was conducted in 18,188 propensity score matched RAMP-DM participants and subjects with diabetes under usual primary care (9,094 subjects in each group). The study endpoints were the first occurrence of coronary heart disease (CHD), stroke, heart failure (HF), total cardiovascular disease (CVD) and all-cause mortality. We constructed multivariable Cox proportional hazard regressions to estimate the association between the RAMP-DM intervention and the first occurrence of study endpoints.

**Results:**

The median follow-up period was 36 months. Three hundred and ninety-nine CVD events occurred in the RAMP-DM group, as compared with 608 in the control group [adjusted hazard ratio, 0.629; 95 % confidence interval (CI) 0.554–0.715; *P* < 0.001]. The total number of all-cause deaths in RAMP-DM group was less than half that of control group (202 vs 552, adjusted hazard ratio, 0.363; 95 % CI, 0.308–0.428; *P* < 0.001). The adjusted hazard ratios of the RAMP-DM group for CHD, stroke, and HF were 0.570 (95 % CI, 0.470–0.691; *P* < 0.001), 0.652 (95 % CI, 0.546–0.780; *P* < 0.001), and 0.598 (95 %CI, 0.446–0.802; *P* = 0.001), respectively.

**Conclusions:**

The RAMP-DM intervention was associated with lower incidences of individual and total cardiovascular complications, as well as all-cause mortality over 3 years follow-up. The encouraging results provided evidence to support that the structured risk-stratification management leading by a multidisciplinary clinical team was an effective approach to reduce future cardiovascular complications in people with diabetes.

Clinical trial registry: NCT02034695, http://www.ClinicalTrials.gov

**Electronic supplementary material:**

The online version of this article (doi:10.1186/s12933-015-0267-3) contains supplementary material, which is available to authorized users.

## Background

The increasing prevalence of diabetes mellitus (DM) posts one of the most challenging health problems worldwide. According to the estimates from the International Diabetes Federation, the number of people with DM is projected to reach 592 million by 2035, a 50 % increase compared to 382 million in 2013 [1]. In China, there are more than 98 million people with DM currently, accounting for about a quarter of the total people with DM in the world [2]. In Hong Kong, one of the most developed regions of China, the prevalence of DM is higher than the reported national average, reaching nearly 10 % [2, 3]. Diabetes has been implicated as the underlying cause of 36 % of all-cause mortality [2] and a leading cause of coronary heart disease and stroke [4]. The annual direct medical cost of diagnosed DM in Hong Kong was estimated to take up 6.4 % of public health care expenditure with the management of diabetes-related complications a major driver of the cost [5].

Although DM increases the risk of cardiovascular disease by 2-5 times [6, 7], the risks of developing diabetic cardiovascular complications vary widely among individuals. Risk prediction algorithms have been developed for cardiovascular complications [8]. Given the increasing number of subjects with DM and the rising disease burden, risk stratification-based management is appealing to avoid complications among high risk cases and allocate limited healthcare resources efficiently. In recent years, guidelines have recommended risk stratification based management [9–11], setting personalized treatment goals based on patients’ individual cardiovascular risks. Personalized management is advocated as a means of translating the evidence from randomized control trials to real-world settings [12]. However, there is a lack of studies on the effectiveness of risk stratification-based personalized management [12].

To implement risk-stratification based management, a multidisciplinary team is required, including nurses, doctors and allied health professionals. The Chronic Care Model advocated by Wagner et al. serves as the conceptual framework for many multidisciplinary diabetes management interventions in primary care [13]. Accumulating evidence shows that multidisciplinary interventions can improve blood glucose control [14–17] and reduce complications in patients with diabetes [18].

To enhance the management of diabetic subjects in primary care setting in Hong Kong, a multidisciplinary risk assessment and management program for patients with diabetes mellitus (RAMP-DM) has been operating in public general out-patient clinics (GOPCs) since August 2009. The details of the intervention and effectiveness of the program at 12 months have been reported [19, 20]. It was found that, compared to the DM subjects under usual care, the RAMP-DM group had significant improvement in their HbA1c levels and predicted cardiovascular risks at 12-months of follow-up [20].

There was a lack of evidence on the long-term effectiveness of multidisciplinary risk-stratification based management programs in Chinese population. This study aimed at evaluating the impact of RAMP-DM on the incidences of diabetic cardiovascular complications and all-cause mortality at 3 years of follow-up. It was hypothesized that RAMP-DM participants would have lower risks of developing the cardiovascular complications and all-cause mortality compared to usual primary care.

## Methods

### Study design

We conducted a prospective cohort study to compare the risks of developing cardiovascular complications and all-cause mortality over 3 years between people with diabetes managed under RAMP-DM and those receiving usual primary care.

### Setting of RAMP-DM

The Hong Kong Hospital Authority, the sole public healthcare provider in Hong Kong, launched the RAMP-DM in August 2009 as a territory-wide primary care service component for people with diabetes in public General Out-patient Clinics (GOPCs). The details of the RAMP-DM program have been reported previously [19]. In brief, the enrolled subjects underwent a comprehensive risk factor screening for diabetes-related complications. Trained Registered Nurses conducted the Nurse Intake Assessment, during which they assessed the screening results and stratified patients into ‘very high’, ‘high’, ‘medium’ and ‘low’ risk groups according to the JADE classification [21]. Then the RAMP-DM subjects were assigned to receive appropriate interventions and education provided by a team of multi-disciplinary healthcare professionals, including Associate Consultants in family medicine, Registered Nurses, Advanced Practice Nurses and allied health professionals (optometrist, dietitian, podiatrist, physiotherapist, etc.) according to their stratified risk level and HbA1c level (Additional file 1: Figure S1). According to patients’ risk levels, some RAMP-DM subjects have annual full risk factors screening and Nurse Intake Assessment, and others have the full assessment every 2–3 years with annual blood test and followed-up by their GOPC doctors.

Subjects under usual primary care continued to be managed by their GOPC doctors without risk assessment and stratification. They were also eligible for referral to allied health professionals at their doctors’ discretion.

### Subjects

The RAMP-DM aimed at covering all people with DM. The inclusion criterion for RAMP-DM is: all subjects with diabetes who are followed up regularly at GOPCs. Up to 31st July 2013, the end date of our study data collection period, there were 147,097 enrolled into RAMP-DM (out of a total of 206,238 patients receiving diabetic care under the primary care service of HA from August 2008 to July 2013). The remaining people with diabetes were continued to be enrolled into RAMP-DM after 31st July 2013, and they served as potential control subjects in this study.

Subjects for this study were identified from the Clinical Management System database of the Hospital Authority. Inclusion criteria for this study are, (1) Age ≥18; (2) Patients with documented International Classification of Primary Care (ICPC-2) codes T89/T90 before baseline dates; (3) Patients with at least one public primary clinics attendance before baseline dates. To evaluate the effectiveness of RAMP-DM in reducing primary diabetes-related complications, we excluded subjects with any pre-existing diabetes-related complications. To reduce potential bias in mortality rates, we further excluded subjects diagnosed with cancer, chronic lung disease and psychological conditions at baseline.

The RAMP-DM cohort comprised subjects with diabetes who were enrolled in RAMP-DM between 1st August 2009 and 31st July 2010. The control group cohort was diabetic subjects who attended GOPC on or before 31st January 2010, and was not enrolled in RAMP-DM by 31st July, 2013. The baseline dates for the RAMP-DM group were their first risk assessment dates. To reduce potential lead-time bias, we set 31st January 2010, the middle date of the baseline dates among RAMP-DM subjects, as the baseline date for the control group. All subjects were observed until a study endpoint or 3 years since their baseline date using the date of their last follow-up as a censor date.

Ethical approval of this study was granted by the Institutional Review Board of the University of Hong Kong/Hospital Authority: Hong Kong West Cluster (UW 10-369), New Territories East Cluster (CRE-2010.543), New Territories West Cluster (NTWC/CREC/1091/12), Kowloon East and Kowloon Central Cluster (KC/KE-10-0210/ER-3), Kowloon West Cluster (KW/EX/10-317 (34–04)), and Hong Kong East Cluster (HKEC-2010-093).

### Propensity score matching

To reduce any selection bias, we matched the subjects in the RAMP-DM and control groups using propensity score matching. This technique pairs individuals based on observable characteristics which indicate a similar probability of receiving treatment (similar propensity score), but one of them received the intervention and the other did not. The propensity score is the conditional probability of receiving the intervention given the observed baseline covariates and it is independent of the outcomes. Propensity score matching is appropriate for studies with a large sample size and many covariates [22]. We generated a propensity score for each patient, and modelled the RAMP-DM intervention as the dependent variable with the baseline covariates as the independent variables. The propensity score matching was conducted using the “psmatch2” STATA package by one-to-one matching without replacement and with a caliper of 0.001, which means the differences of propensity scores for each matched pair was within 0.001. The unmatched control subjects were discarded.

The baseline covariates for developing the propensity score were (1) demographic characteristics, including age, sex, whether on comprehensive social security assistance; (2) clinical parameters, including smoking status, duration of diabetes, HbA1c, low-density lipoprotein cholesterol (LDL-C), total cholesterol (TC), high-density lipoprotein cholesterol (HDL-C), triglyceride systolic blood pressure (SBP), diastolic blood pressure (DBP), body mass index (BMI), and estimated glomerular filtration rate; (3) treatment modality, including oral glucose-lowering drugs, anti-hypertensive drugs, lipid-lowering drugs and insulin; and (4) comorbidities, measured by the Charlson comorbidity score [23].

### Study endpoints

The endpoint for this study was the time to first occurrence of a major diabetes-related complication including (1) coronary heart disease (CHD), (2) stroke, (3) heart failure (HF), (4) a composite of the former three cardiovascular diseases (CVD) and (5) death from any cause. The incidence of diabetes-related complications was identified by the International Classification of Diseases, Ninth Edition, Clinical Modification and ICPC-2 codes from the Clinical Management System of the Hospital Authority (Table 1). The death cases were identified from death registration data.

**Table 1:** ICD-9CM, ICPC-2 codes for diabetic macrovascular complications

Disease	ICPC-2 codes	ICD-9-CM codes
Coronary heart disease (CHD)	K74–K76	410.00–410.92; 411.0–411.89; 412; 413.0–413.9; 414.0–414.9; 798.1–798.9
Heart failure	K77	428.0–428.9
Stroke	K89–K91	430; 431; 432.0–432.9; 433.00–433.91; 434.00–434.91; 435.0–435.9; 436; 437.0–437.9; 438.0–438.9

*ICD-9-CM* International classification of diseases, ninth edition, clinical modification; *ICPC-2* international classification of primary care.

### Data analysis

We used the independent t test or Chi squared test, as appropriate, to compare the demographic and clinical parameters between the RAMP-DM and control groups at baseline and the end of follow-up. The cumulative incidence rates for each type of diabetic complication and all-cause mortality are reported. We constructed the 95 % confidence intervals of the incidence rates based on the assumption that the observed incident events followed a Poisson distribution.

For each of the study endpoints, the Kaplan–Meier method was used to estimate the survival curves and the log-rank test was used to compare the between group differences. To estimate the magnitude of differences in endpoints, multivariable Cox proportional hazards regression models were employed to explore the effects of RAMP-DM on the dependent variables of each endpoint, adjusting for all the baseline covariates. The hazard ratio (HR) with 95 % confidence interval were reported for each of the endpoints. The predictive accuracy of each regression model was evaluated using Harrell’s discrimination C-index, ranging from 0 to 1. A value of 0.5 indicates the model does not have predictive discrimination ability, and values of 0 or 1 indicate perfect ability to discriminate subjects [24]. As the interventions in RAMP-DM groups were stratified by subjects’ HbA1c levels, we further explored the effects of RAMP-DM in preventing diabetic complications in three subgroups, i.e. HbA1c <7 % (53 mmol/mol), 7 %≤ HbA1c <8.5 % (69 mmol/mol), and HbA1c ≥8.5 %.

Intention to treat analysis was adopted.

We performed all the statistical analyses using STATA Version 13.0 (StataCorp LP. College Station, Texus, US), and *P* value less than 0.05 was considered as statistically significant.

## Results

### Baseline characteristics

**Fig. 1:**
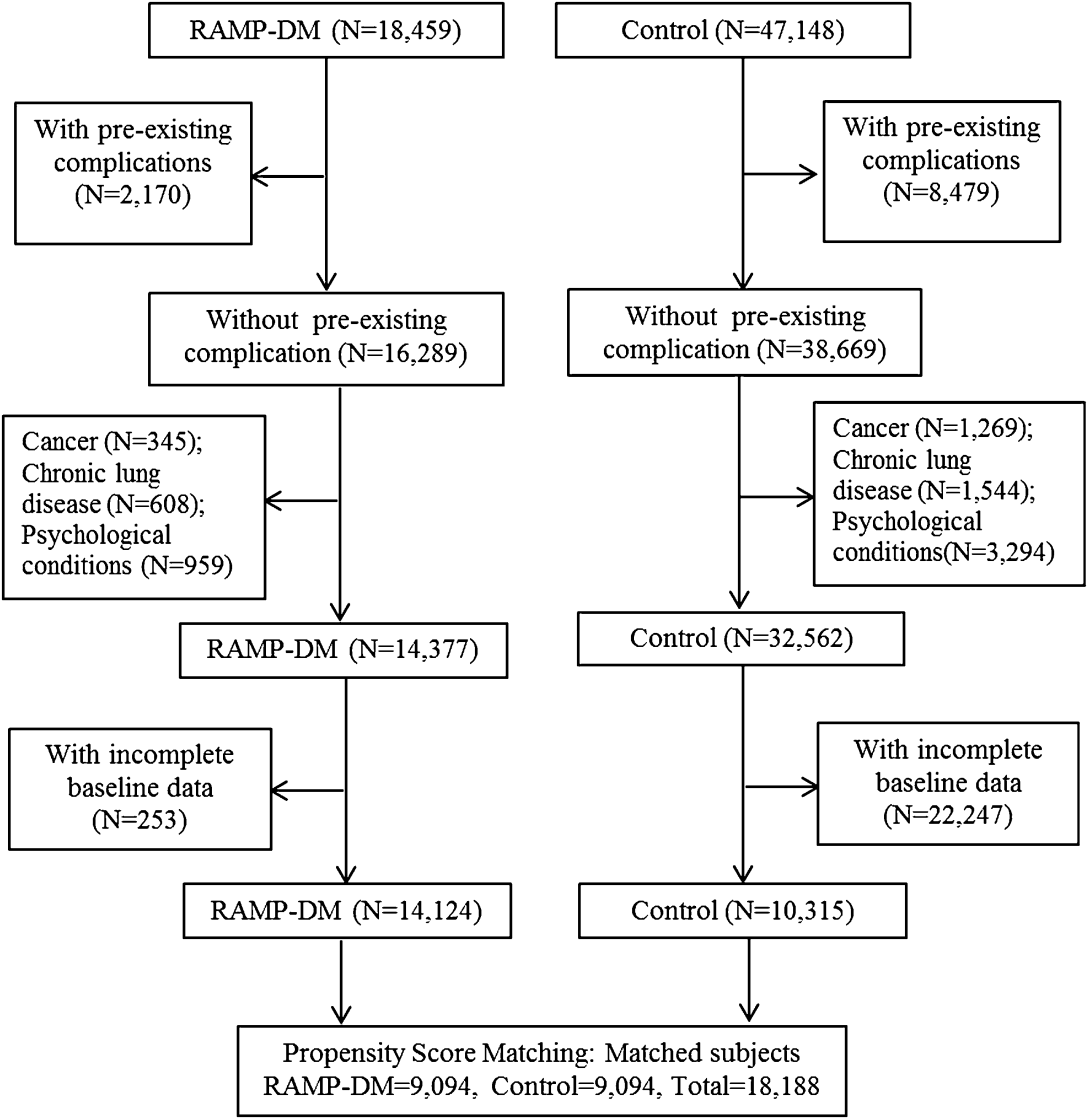
Flow chart of subjects matching. *RAMP-DM* risk assessment and management programme for patients with diabetes mellitus.

From 1st Aug 2009 to 31st July 2010, a total of 18,459 diabetic subjects under primary care were enrolled in RAMP-DM (Fig. 1). We identified 47,148 potential control subjects met the inclusion criteria for the study. Subjects with any existing diabetes-related complications were excluded in each group, giving 16,289 and 38,669 subjects in RAMP-DM and control groups respectively. Limiting the eligibility to subjects without prior diagnosis of cancer, chronic lung disease and psychological conditions in both groups reduced the sample to 14,377 and 32,562 in RAMP-DM and control groups respectively. We further excluded 22,500 cases (253 in RAMP group and 22,247 in control group) with incomplete baseline data, leaving 14,124 RAMP-DM subjects and 10,315 control subject. To reduce selection bias, we further refined the study sample using propensity score matching. The final matched sample for this study comprised 9,094 RAMP-DM subjects and 9,094 control subjects.

The comparison of baseline characteristics between the two groups is shown in Table 2. At baseline, all the demographic, clinical parameters and treatment modality of the patients were not significantly different between the two groups. The average age of the two cohorts was 64, and around 87 % subjects were on oral glucose-lowering drugs. At the end of follow-up, the RAMP-DM subjects showed significantly lower HbA1c (7.13 vs 7.25 %, *P* < 0.001) and SBP (130.12 vs 132.35 mm Hg, *P* < 0.001). Regarding the treatment modalities, the RAMP-DM group showed higher percentages of patients on all the four types of drugs (glucose-lowering drugs, anti-hypertensive drugs, lipid-lowering drugs and insulin) than the control group.

**Table 2:** Basic characteristics at baseline and 3 years

	At baseline			At the end of follow-up		
	RAMP (N = 9,094)	Control (N = 9,094)	*P* value^a^	RAMP (N = 8,892)	Control (N = 8,542)	*P* value^a^
	Mean ± SD or N (%)	Mean ± SD or N (%)		Mean ± SD or N (%)	Mean ± SD or N (%)	
Socio-demographic						
Age (year)	64.23 ± 11.05	64.29 ± 11.96	0.751			
Female	4,713 (51.8 %)	4,774 (52.5 %)	0.365			
On CSSA	1,293 (14.2 %)	1,340 (14.7 %)	0.322			
Clinical measures						
Duration of DM (year)	8.31 ± 6.75	8.42 ± 6.15	0.258			
Current smoker	927 (10.2 %)	906 (10.0 %)	0.605	346 (9.0 %)	235 (8.6 %)	0.651
BMI (kg/m^2^)	25.33 ± 3.74	25.33 ± 3.90	0.900	25.07 ± 3.79	25.11 ± 3.92	0.540
SBP (mm Hg)	135.41 ± 17.05	135.45 ± 16.56	0.865	130.12 ± 14.68	132.35 ± 15.51	<0.001
DBP (mm Hg)	75.11 ± 10.34	75.08 ± 9.77	0.828	71.60 ± 10.26	73.23 ± 9.72	<0.001
HbA1c (%)	7.24 ± 1.23	7.24 ± 1.24	0.775	7.13 ± 1.09	7.25 ± 1.26	<0.001
TC (mmol/L)	5.08 ± 0.94	5.08 ± 0.95	0.976	4.43 ± 0.82	4.49 ± 0.86	<0.001
HDL-C (mmol/L)	1.22 ± 0.32	1.22 ± 0.32	0.504	1.28 ± 0.34	1.31 ± 0.35	<0.001
LDL-C (mmol/L)	3.13 ± 0.82	3.14 ± 0.83	0.487	2.51 ± 0.69	2.55 ± 0.72	<0.001
Triglyceride (mmol/L)	1.64 ± 1.10	1.64 ± 1.05	0.923	1.43 ± 0.87	1.43 ± 0.97	0.874
eGFR (ml/min/1.73 m^2^)	81.68 ± 20.81	81.68 ± 19.43	0.983	80.88 ± 22.92	81.02 ± 22.51	0.725
Charlson comorbidity score^b^	0.04 ± 0.26	0.04 ± 0.26	0.651	0.07 ± 0.35	0.08 ± 0.38	0.069
Percentage reaching treatment target						
SBP <130 mmHg	37.61 %	36.83 %	0.276	50.31 %	45.45 %	<0.001
DBP <80 mmHg	66.49 %	67.39 %	0.202	78.39 %	74.45 %	<0.001
HbA1c <7 %	48.37 %	47.36 %	0.172	52.10 %	48.81 %	<0.001
LDL-C <2.6 mmol/L	26.57 %	27.38 %	0.218	59.18 %	58.46 %	0.384
Treatment modality						
On glucose-lowering drugs	7,943 (87.3 %)	7,929 (87.2 %)	0.755	7,999 (90.0 %)	7,143 (83.6 %)	<0.001
On anti-hypertensive drugs	6,637 (73.0 %)	6,673 (73.4 %)	0.547	7,112 (80.0 %)	6,493 (76.0 %)	<0.001
On lipid-lowering drugs	1,189 (13.1 %)	1,225 (13.5 %)	0.431	4,551 (51.2 %)	3,903 (45.7 %)	<0.001
On insulin	105 (1.2 %)	130 (1.4 %)	0.101	534 (6.0 %)	386 (4.5 %)	<0.001

*BMI* body mass index, *CSSA* comprehensive social security assistance, *DBP* diastolic blood pressure, *DM* dibetes mellitus, *eGFR* estimated glomerular filtration rate, *HbA1c* hemoglobin A1c, *HDL-C* high density lipid cholesterol, *LDL-C* low density lipid cholesterol, *SBP* systolic blood pressure and *TC* total cholesterol.

^a^Significant differences (*P* < 0.05) between groups by independent t test or by Chi-square test, as appropriate.

^b^Add up the comorbidity component score in Charlson comorbidity index.

### Observed incidence of diabetes-related complications and all-cause mortality

Table 3 shows the observed number of the first diagnoses of each type of diabetes-related complication and the incidence rates over a median follow-up period of 36 months among 9,094 subjects in each group. More than 25,000 person-years of observation were available for each of the study endpoints in both groups. The RAMP-DM group had lower incidence rates for all the primary and secondary endpoints with 399 and 608 CVD events in the RAMP-DM and control groups respectively. Stroke was the most prevalent CVD in both groups (205 and 309 stroke events in RAMP-DM and control group, respectively). Over the observation period, 552 death cases occurred in the control group, which was more than twice that in the RAMP-DM group (202 cases).

**Table 3:** Number of diabetic macrovascular complication and all-cause death at a median follow-up of 36 months

	Cumulative incidence		Incidence rate (cases/100 person-years)		
	No. of events	Rate (%)	Estimate	95 % CI*	Person-years
RAMP-DM group (N = 9,094)					
CVD	399	4.39	1.495	(1.352, 1.650)	26,681
CHD	170	1.87	0.631	(0.540, 0.733)	26,938
Stroke	205	2.25	0.763	(0.662, 0.874)	26,882
Heart Failure	72	0.79	0.266	(0.208, 0.335)	27,040
All-cause mortality	202	2.22	0.745	(0.646, 0.856)	27,101
Control group (N = 9,094)					
CVD	608	6.69	2.420	(2.231, 2.620)	25,127
CHD	280	3.08	1.099	(0.974, 1.235)	25,482
Stroke	309	3.40	1.216	(1.084, 1.359)	25,418
Heart failure	125	1.37	0.487	(0.405, 0.580)	25,660
All-cause mortality	552	6.07	2.144	(1.968, 2.330)	25,752

*RAMP-DM* multi-disciplinary risk assessment and management-diabetes mellitus. *CVD* cardiovascular disease, *CHD* coronary heart disease and *CI* confidence interval.

* The 95 % CI was constructed based on Poisson distribution.

**Fig. 2:**
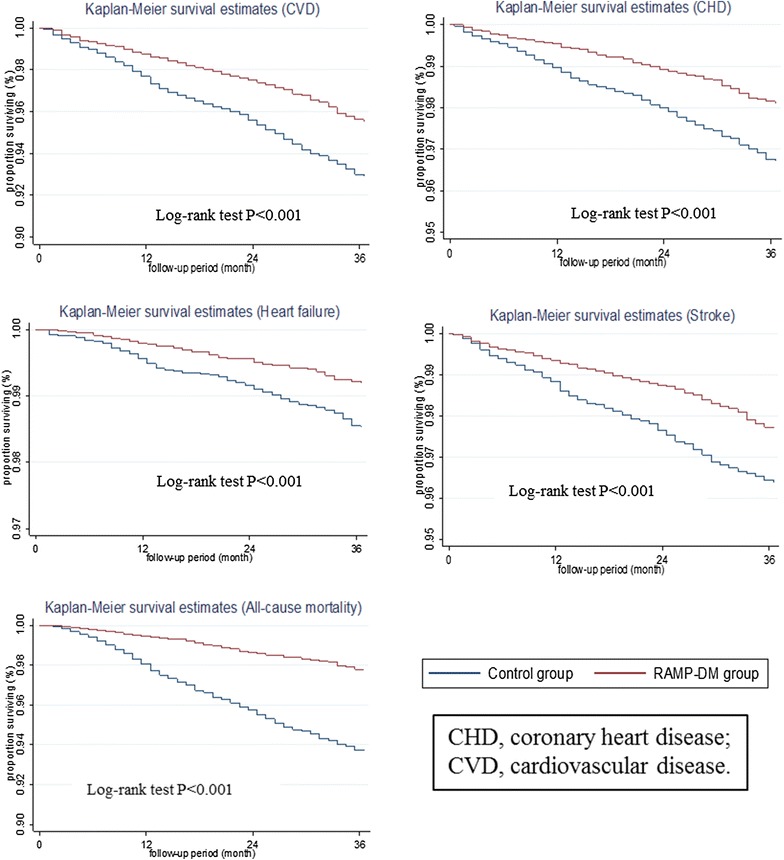
Kaplan-Meier survival curves. *CVD* cardiovascular diseases and *CHD* coronary heart disease.

The Kaplan–Meier survival curves for each study end point are shown in Fig. 2. For all the cardiovascular complications and all-cause mortality, the RAMP-DM group showed lower incidence rates from the beginning of the observation period and the differences became larger over the follow-up period. The log-rank test showed the RAMP-DM group had significantly lower risks of all the studied complications and all-cause mortality than the control group.

### Multivariable Cox regression models

We constructed multivariable Cox regression models to evaluate the hazard ratios (HR) between RAMP-DM and control groups for each study endpoint adjusted for all the baseline covariates. As shown in Table 4, compared to the control group, the RAMP-DM group significantly reduced the incidence of total CVD (HR: 0.629, 95 % CI 0.554–0.715, *P* < 0.001) and also had significant lower risks of developing CHD (HR: 0.570, 95 % CI 0.470–0.691, *P* < 0.001), stroke (HR: 0.652, 95 % CI 0.546–0.780, *P* < 0.001) and heart failure (HR: 0.598, 95 % CI 0.446–0.802, *P* = 0.001). The all-cause mortality was significantly lower in the RAMP-DM group (HR: 0.363, 95 % CI 0.308–0.428, *P* < 0.001).

**Table 4:** Multivariable Cox proportional hazard regression in all endpoints

	RAMP-DM vs Control				Harrell’s C-statistic
	HR^†^	SE	95 % CI	P value	
RAMP-DM subjects vs control subjects (all subjects, N = 18,188)					
CVD	0.629	0.041	(0.554, 0.715)	<0.001	0.75 (0.73,0.76)
CHD	0.570	0.056	(0.470, 0.691)	<0.001	0.74 (0.72,0.76)
Stroke	0.652	0.059	(0.546, 0.780)	<0.001	0.76 (0.74,0.78)
Heart failure	0.598	0.090	(0.446, 0.802)	0.001	0.87 (0.85,0.90)
All-cause mortality	0.363	0.030	(0.308, 0.428)	<0.001	0.82 (0.80,0.83)
RAMP-DM subjects vs control subjects (baseline HbA1c <7 %, N = 8,540)					
CVD	0.592	0.055	(0.494, 0.711)	<0.001	0.74 (0.72,0.76)
CHD	0.553	0.075	(0.423, 0.722)	<0.001	0.75 (0.71,0.78)
Stroke	0.553	0.075	(0.425, 0.720)	<0.001	0.75 (0.72,0.78)
Heart failure	0.680	0.136	(0.459, 1.006)	0.054	0.88 (0.85,0.91)
All-cause mortality	0.415	0.046	(0.335, 0.515)	<0.001	0.81 (0.79,0.83)
RAMP-DM subjects vs control subjects (baseline HbA1c 7–8.5 %, N = 7,047)					
CVD	0.703	0.074	(0.572, 0.864)	0.001	0.75 (0.73,0.77)
CHD	0.650	0.108	(0.470, 0.899)	0.009	0.75 (0.71,0.79)
Stroke	0.779	0.111	(0.590, 1.029)	0.079	0.77 (0.74,0.80)
Heart failure	0.455	0.124	(0.267, 0.776)	0.004	0.88 (0.84,0.92)
All-cause mortality	0.292	0.043	(0.219, 0.389)	<0.001	0.83 (0.80,0.85)
RAMP-DM subjects vs control subjects (baseline HbA1c ≥8.5 %, N = 2,257)					
CVD	0.576	0.109	(0.397, 0.836)	0.004	0.79 (0.75,0.83)
CHD	0.450	0.132	(0.254, 0.799)	0.006	0.75 (0.68,0.81)
Stroke	0.624	0.164	(0.373, 1.043)	0.072	0.85 (0.81,0.88)
Heart failure	0.664	0.294	(0.279, 1.581)	0.355	0.86 (0.78,0.94)
All-cause mortality	0.303	0.085	(0.175, 0.525)	<0.001	0.84 (0.79,0.88)

Adjusted for the socio-demographic and clinical characteristics.

*AIC* Akaike information criterion, *BIC* Bayesian information criterion, *CHD* coronary heart disease, *CVD* cardiovascular disease, *HR* hazard ratio, *SE* standard error and *HbA1c* hemoglobin A1c.

^†^HR >1 indicates greater risk for endpoints.

### Sub-group analysis

The results of sub-group analysis are shown in Table 4. We found that the RAMP-DM group had significantly lower risks of developing CHD, total CVD and of all-cause mortality in all three sub-groups. The decrease in total CVD risk in the RAMP-DM group in the 7 %≤ HbA1c <8.5 % group was lower than the other two sub-groups (HbA1c <7 % and HbA1c ≥8.5 %), whereas the risk of heart failure decreased most in this group.

## Discussion

To the best of our knowledge, this is the first study exploring the long-term effects of a multidisciplinary risk-stratified management program in reducing diabetic cardiovascular complications and all-cause mortality in Chinese population. This propensity score matched cohort study found that RAMP-DM was significantly associated with decreases in the risks of all the cardiovascular complications and all-cause mortality. Compared to subjects under usual primary care, subjects enrolled in RAMP-DM had 37.1 and 63.7 % lower risks of developing total CVD and all-cause mortality, respectively. Also, the RAMP-DM group had a lower incidence of all three components of CVD, with adjusted HRs of 0.570 (95 % CI 0.470 to 0.691, *P* < 0.001), 0.652 (95 % CI 0.546 to 0.780, *P* < 0.001) and 0.598 (95 % CI 0.446 to 0.802, *P* = 0.001) for CHD, stroke and heart failure respectively. The substantial reduction in the incidence of all-cause mortality in RAMP-DM group might be mainly attributed to the significant decreases in life-threatening diabetic cardiovascular complications.

Sub-group analysis showed that RAMP-DM was associated with decreased risks of total CVD, CHD and all-cause mortality in all three sub-groups. In the sub-group of subjects with HbA1c ≥8.5 %, the risks of stroke and heart failure were similar between RAMP-DM and control groups. It is possible that for subjects with higher HbA1c, doctors tended to give them more intensive intervention, no matter whether they were enrolled in RAMP-DM or not, and patients under usual care were eligible to be referred to some services in the RAMP-DM intervention package, like care from allied health professionals, which might bias the effects of RAMP-DM towards null. The relatively small number of subjects (2,257 out of 18,188) in this sub-group might also limit the power to detect a significant effect although the results favored the RAMP-DM group.

Previously, we reported a pilot study in a sample of 1,072 pairs of RAMP-DM and control subjects at 12 months follow-up [20]. We found that the RAMP-DM group showed improvement in surrogate effectiveness measures including HbA1c level and predicted cardiovascular risks. Although the numbers of observed CVD events were small due to a short follow-up period and small sample size, the observed CVD event rate in the RAMP-DM group was significantly lower (RAMP-DM vs control: 1.21 vs 2.89 %, *P* = 0.003) at 12 months follow-up. The current study confirmed the favorable findings for the RAMP-DM group in avoidance of cardiovascular complications using a more representative sample and a longer follow-up period.

Previous studies on short-term effectiveness of risk-stratification based intervention were conducted in U.S. [25] and U.K. [26]. Both studies reported the increase in the percentages of subjects reaching target HbA1c, blood pressure in the intervention group. However, long-term effectiveness of the intervention on cardiovascular events was not reported. In Asia, attempt for the risk stratification management was made by the Joint Asia Diabetes Evaluation Program (JADE) [27]. Clinicians can access a web-based comprehensive risk stratification model using an electronic portal. During 2007–2009, 3,687 people with diabetes across seven Asian countries, including Hong Kong, were enrolled [21]. The implementation of the structured care and effectiveness of this care model compared to usual care is not clear.

Our study reported the long-term effectiveness of the risk-stratification management for patients with DM. We observed significant lower incidences of all cardiovascular complications in the RAMP-DM group, whereas the between group differences of HbA1c was modest (7.13 ± 1.09 % vs 7.25 ± 1.26 %, *P* < 0.001) at three-year follow-up. Although it is reported that an increase in HbA1c level is significantly associated with the incidence of coronary heart disease over 6 years follow-up [28], intensive blood glucose control centered on drug interventions did not necessarily lead to success in preventing future cardiovascular events. The Preterax and Diamicron Modified Release Controlled Evaluation (ADVANCE) trial showed that over 5 years of follow-up, the intensive control group achieved lower HbA1c than the control group (6.5 vs 7.3 %), but it failed to reduce the risk all the cardiovascular complications [29]. The intensive glucose control trial in the United Kingdom Prospective Diabetes Study (UKPDS) found that after 10 years follow-up, although the intensive glucose control had 11 % reduction in HbA1c (7.0 vs 7.9 %), they did not observed significant risk reduction in any cardiovascular complications [30]. Interestingly, the post-trial 10 years follow-up found decreased risks of myocardial infarction and all-cause death although the two groups converged to similar HbA1c in the follow-up period [31].

These contradictory results encourage exploration of alternative approaches to long-term management of people with diabetes. The Steno-2 study implemented a multifactorial intervention including a combination of medications and focused behavior modification. It was reported that the intervention group had lower risks of cardiovascular events (HR 0.41) over 13.3 years follow-up compared to the conventional care group [32]. A physician-led structured diabetes management program in Germany also showed lower incidences of MI (risk ratio 0.75) and stroke (risk ratio 0.80) among the intervention group over 4 years follow-up. This program involved education and structured evidence-based care by physicians [33].

The remarkable reduction in the incidences of cardiovascular complications and all-cause mortality in the RAMP-DM group might result from several reasons. First, structured risk assessment improved adherence to recommendation on annual assessment to detect reversible risk factors early, e.g. hypertension and hyperlipidemia, so that timely interventions could be given to prevent further deterioration. Second, significantly higher proportions of subjects in the RAMP-DM group were treated with glucose-lowering drugs, insulin, anti-hypertensive drugs and lipid-lowering drugs, which suggested that the doctors might have managed the patients more intensively after knowing the risk stratification by RAMP-DM. Third, the risk stratification might also have positive impact on improving patients’ consciousness of health and motivating them to change lifestyles. Physical activity was found to decrease the risk of cardiovascular morality in DM patients [34]. Fourth, the multidisciplinary RAMP-DM team provided more education, e.g. the smoking cessation, about complications prevention, providing patients with additional treatment. Fifth, RAMP-DM also led to significant decreases in HbA1c, blood pressure and LDL-C compared to the control group, which could all lower the complication risks. These changes could all contributed to the impressive effectiveness of the RAMP-DM intervention, which was consistent with the remarkable findings of multifactorial Steno-2 study [32] and the multidisciplinary care in Germany [33]. However, longer term follow-up is required to valid the effectiveness of RAMP-DM over a longer time-span.

### Strength and limitations of this study

This population-based prospective comparative effectiveness study had several strengths. First, we sampled the study subjects from the Clinical Management System of the Hospital Authority, which recorded data on all the people with diabetes managed in the public healthcare sector. This population-based sample was highly representative of the Hong Kong population with diabetes. Second, we included comprehensive covariates to develop propensity score matching for the two groups. The observed risk factors that might affect the incidence of diabetic cardiovascular complications were included. The two groups were well matched at baseline and we further adjusted all the covariates during multivariable Cox regression to minimize any possible bias. Third, we conducted this comparative effectiveness study using primary care data, providing evidence, if real, can be applied in a real-world setting. Fourth, we used an intention-to-treat analysis, giving a more conservative estimate of the effectiveness of RAMP-DM.

An important limitation of this study is that we could not carry out a randomized study therefore unobserved potential confounders might affect the results, although we have minimised this as much as we can. Second, the control subjects in our study were selected from those who were either had not been introduced to the RAMP-DM or refused to join the program. There was a possibility that the control subjects were less health conscious than the RAMP-DM subjects, which could partly contribute to their worse outcomes. Third, not all the RAMP-DM subjects were included in the analysis due to missing data at baseline and some subjects were further excluded due to unavailable matched control pairs. Fourth, we identified the occurrence of cardiovascular complications by clinical diagnosis codes. There was a possibility of under-diagnoses or coding misclassification errors. Fifth, 3 years are not long enough to project the longer term benefits of RAMP-DM. We will continue to observe the effects of RAMP-DM over a longer time-span.

## Conclusion

This prospective comparative effectiveness study in a pragmatic primary care setting found that RAMP-DM was associated with decreased incidences of total CVD, CHD, stroke, heart failure, and all-cause mortality among diabetic subjects without pre-existing complications over 3 years of follow-up. These findings provide imperative translational evidence of the effectiveness of multidisciplinary risk-stratification based management for Chinese people with DM. Further study on the longer term effects of RAMP-DM in preventing diabetic cardiovascular complications will be conducted with a longer follow-up period.

## Additional file

Additional file 1:
**Figure S1.** The patient management flowchart of RAMP-DM. Legends: APN, Advanced Practice Nurse; AC, Associate consultant.

## Abbreviations

ADVANCE: Preterax and Diamicron Modified Release Controlled Evaluation
CVD: cardiovascular disease
DBP: diastolic blood pressure
DM: diabetes mellitus
GOPCs: general out-patient clinics
HF: heart failure
HbA1c: hemoglobin A1c
HR: hazard ratio
ICD-9-CM: international classification of diseases, ninth edition
ICPC-2: international classification of primary care
JADE: joint Asia diabetes evaluation program
LDL-C: low density lipoprotein—cholesterol
RAMP-DM: multidisciplinary risk assessment and management program for patients with diabetes mellitus
SBP: systolic blood pressure
UKPDS: United Kingdom Prospective Diabetes Study
