# Supplementary material for: Long-term effects of the multidisciplinary risk assessment and management program for patients with diabetes mellitus (RAMP-DM): a population-based cohort study
Source: Cardiovasc Diabetol. 2015 Aug 14;14:105. doi: 10.1186/s12933-015-0267-3 (PMC4535739; doi:10.1186/s12933-015-0267-3)

If Applicable

Patients with DM

Risk factors screening and risk stratification

Dietitian

Podiatrist

Physiotherapist

Ophthalmologist

Wound Clinic

Smoking Counselling and Cessation Service


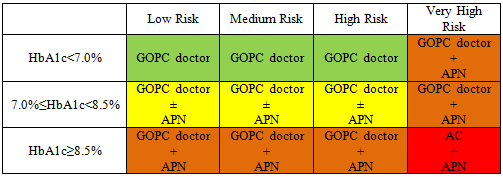

Supplement: Additional file 1: — Figure S1. The patient management flowchart of RAMP-DM. Legends: APN, Advanced Practice Nurse; AC, Associate consultant. [file 12933_2015_267_MOESM1_ESM.docx]
